# Stathmin 1 expression predicts prognosis and benefits from adjuvant chemotherapy in patients with gallbladder carcinoma

**DOI:** 10.18632/oncotarget.19625

**Published:** 2017-07-27

**Authors:** Xiaobo Bo, Jie Wang, Qiang Fu, Yueqi Wang, Houbao Liu, Jiejie Xu

**Affiliations:** ^1^ Department of General Surgery, Zhongshan Hospital, Fudan University, Shanghai, China; ^2^ Department of Biochemistry and Molecular Biology, School of Basic Medical Sciences, Fudan University, Shanghai, China

**Keywords:** gallbladder carcinoma, stathmin, prognosis, adjuvant chemotherapy, overall survival

## Abstract

**Background:**

Abnormal expression of Stathmin 1(STMN1) plays an important role in the proliferation and migration of gallbladder carcinoma (GBC). The purpose of current study is to investigate the prognostic significance of STMN1 in GBC patients after surgery.

**Methods:**

STMN1 expression was evaluated with immunohistochemistry (IHC) on tissue microarrays from 70 GBC patients from a single institution between 2009 and 2013. The correlation between STMN1 expression and clinicopathological profiles and the prognosis was statistically inspected.

**Results:**

High expression of STMN1 in tumoral tissue was associated with poor tumor differentiation (*P*<0.001), lymph node metastasis (*P*=0.028), advanced TNM stage (*P*=0.011) and short overall survival (*P*<0.001). Cox multivariate analysis identified the STMN1 expression as an independent prognostic factor. Integrating STMN1 expression with current TNM staging system generate a better clinical predictive model for GBC. Moreover, the postoperative adjuvant chemotherapy (ACT) showed significant benefit in TNM III- IV stage patients with low STMN1 expression.

**Conclusion:**

STMN1 might be an independent adverse prognostic factor in GBC patients after surgery, which could be combined with TNM staging system to improve the predictive accuracy for overall survival. Low expression of STMN1 stratified a subgroup of advanced GBC patients who could benefit from ACT.

## INTRODUCTION

GBC, the most common biliary tract cancer, has a very poor prognosis because of its highly lethal nature[1]. The incidence of GBC varies by geographic region. The incidence of GBC in China, Thailand, and northern India is obviously higher than that in Europe and America[2, 3]. Radical resection is currently the main treatment option for GBCs[4, 5]. Unfortunately, due to the nonspecific symptoms and highly invasive character of GBC, only a minority of patients are candidates for curative resection at the time of diagnosis[4, 6]. In current, the commonly used TNM staging system is inadequate to predict accurate clinical outcomes of GBCs. Moreover, the benefit of ACT for advanced GBC patients remains unfavorable[7, 8]. Therefore, exploration of new biomarkers might provide a better prognostic prediction model and the guidance for advanced GBC patients with treatment of ACT.

STMN1, belonged to a family of microtubule-destabilizing protein, has been reported to be overexpressed in various malignant cancers, such as leukemia, ovarian, gastric cancer, colon, lung, and prostate cancers[9–11]. Recently, there are some studies revealing that STMN1 could promote cell proliferation, mobility and metastasis[12, 13]. High STMN1 expression in tumor tissue is associated with increased invasion and lymph node metastasis and poor survival[14]. A recent research proved that suppressing STMN1 expression inhibited cell proliferation, migration and invasion of GBC cell lines. Silencing of STMN1 caused G2/M arrest and apoptosis of GBC cell lines[15]. However, the relationship between STMN1 expression and clinical prognosis remains unclear.

In this study, we evaluated the correlation between STMN1 expression and the clinical pathological characteristics and overall survivals. Furthermore, it is investigated that the role of STMN1 as a predictive biomarker for advanced GBC patients who received ACT.

## RESULTS

### Intratumoral expression of STMN1 and its association with clinicopathological characteristics

**Figure 1:**
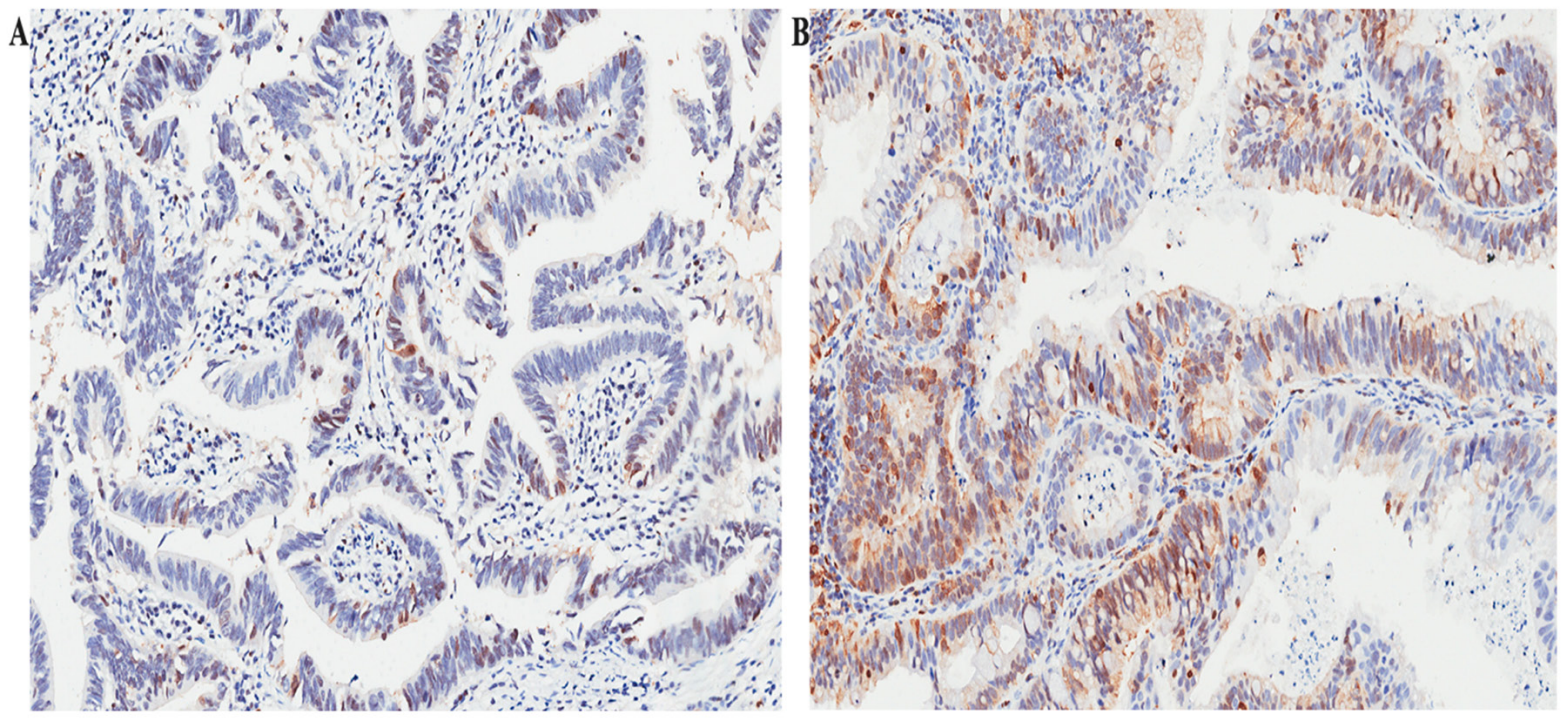
Representative images for STMN1 expression in GBC GBC tissue with low STMN1 expression **(A)** and high STMN1 expression **(B)** magnification 200×.

The positive staining of STMN1 was observed in the cytoplasm and/or on the membrane of neoplastic epithelial (Figure 1A, 1B). The cutoff point of STMN1 expression was 90, which was determined by the method of minimum p value with the X-tile software. Thus, 44 patients were separated into STMN1 low expression subgroup and 26 patients were separated into the STMN1 high expression subgroup. The prognosis was evaluated based on the expression of STMN1 in GBC tissues. The correlation between clinical pathological characteristics and STMN1 expression were shown in Table 1. These findings indicated that STMN1 overexpression in GBC tissues was positively associated with lymph node metastasis (p=0.028), distant metastasis (p=0.029), histological differentiation (p=0.029) and TNM stage (P=0.011). No significant association between STMN1 and other clinical pathological factors was found.

**Table 1:** Associations between STMN1 expression and clinical pathological characteristics in patients with GBC

Variable	Stathmin expression			*P*
	Total	Low	High	
Age(y)				0.263
Mean±SD	68.77±14.64	63.30±10.32	66.35±11.95	
Gender				0.557
Male	19	13	6	
Female	51	31	20	
T classification				0.555
1	2	2	0	
2	10	8	2	
3	41	26	15	
4	17	8	9	
N classification				0.028
Absent	39	29	10	
Present	20	10	10	
Not available	11	5	6	
Distant metastasis				0.029
Absent	14	5	9	
Present	56	39	17	
Differentiation				<0.001
G1	3	3	0	
G2	25	19	6	
G3	29	13	16	
G4	13	9	4	
TNM stage				0.011
I	2	2	0	
II	5	5	0	
III	35	23	12	
IV	28	14	14	
Adjuvant chemotherapy				0.236
Yes	32	23	9	
No	38	21	17	

### Prognostic value of STMN1 Expression in GBC

**Figure 2:**
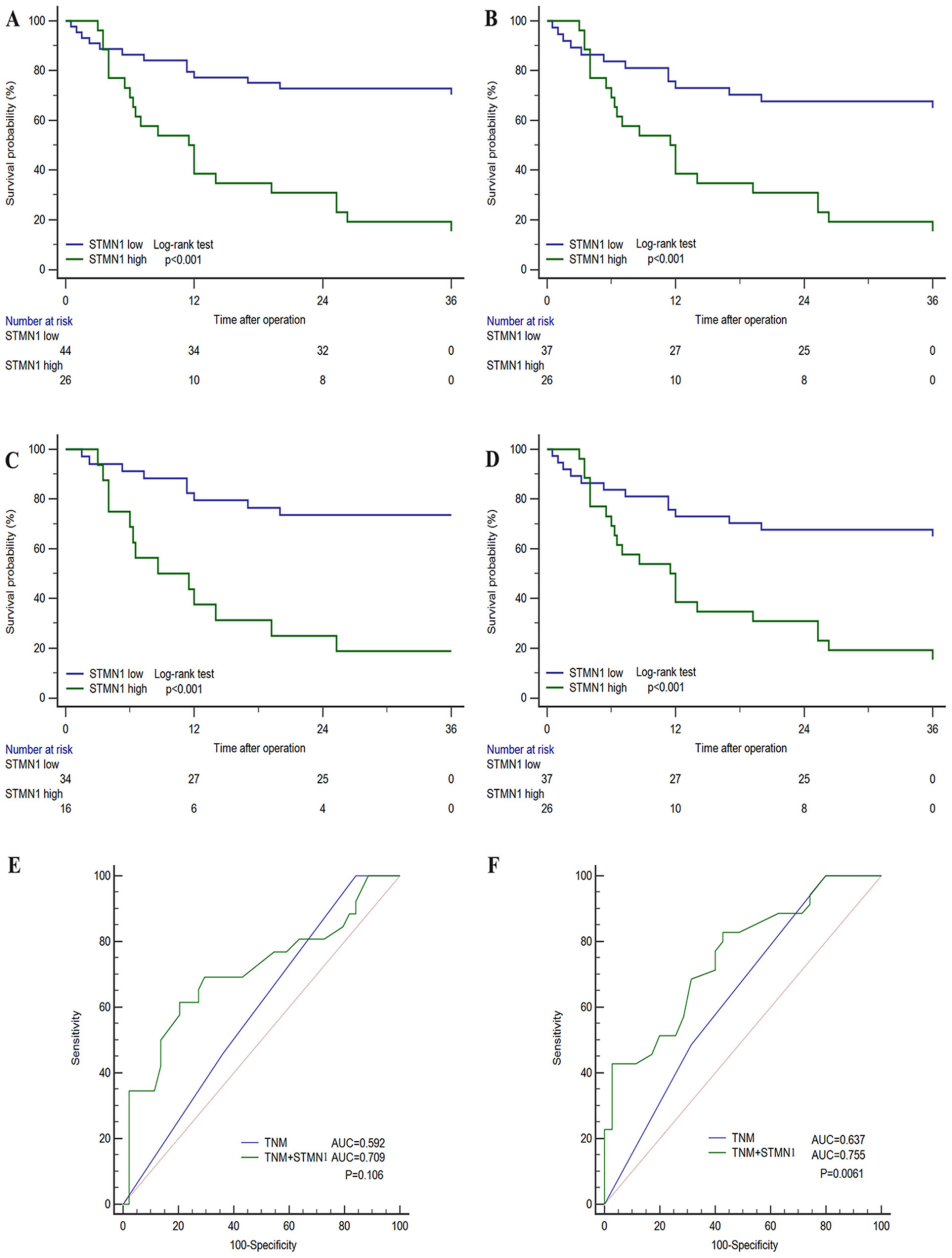
Subgroup Kaplan-Meier analyses of overall survival to assess prognostic value of STMN1 in GBC patients **(A)** all patients. **(B)** patients with TNM III- IV stage tumor. **(C)** patients with N0 stage tumor. **(D)** patients with G3-4 differentiated tumor. **(E)** and **(F)** ROC analysis of the prognosis sensitivity and specificity for overall survival by TNM stage model and TNM stage/STMN1 expression model in all patients at 12 months and 36 months, respectively.

Kaplan-Meier method with log-rank test was performed to analysis the relationship between STMN1 expression and overall survival in the two subgroups. As shown in Figure 2, patients with low STMN1 expression were prone to longer OS. High expression of STMN1 has a poorer prognosis compared with low expression (Figure 2A, p<0.001). To investigate the prognostic significance of STMN1 expression based on different clinical pathological characteristics, we performed subgroup analysis in patients with different TNM stage, depth of tumor invasion, lymph node metastasis and tumor differentiation, respectively. As a result, significances were observed in TNM III- IV stage (Figure 2B, p<0.001), lymph nodal metastasis (Figure 2C, p<0.001), G3-4 differentiation (Figure 2D, P=0.031). The T classification (p<0.001), N classification (p=0.008), distant metastasis (p=0.026), differentiation (p=0.009), STMN expression (P<0.001) and ACT (P=0.005) were identified as prognostic factors by univariate Cox regression model. To further explore the predictive value of STMN1 precisely, the factors whose p value was <0.05 according to univariate Cox regression were selected into multivariate Cox regression analysis. As shown in Table 2, T classification (p=0.034, HR2.28, 95% CI1.06-4.91), STMN1 expression (p=0.028, HR1.11, 95% CI1.01-1.20) and ACT (P=0.034, P=0.43, 95% CI 0.19-0.94) were statistically identified to independent prognostic factors for overall survival for patients with GBC.

**Table 2:** Univariate and multivariate Cox regression analyses for overall survival

Variables	Univariate analysis		Multivariate analysis	
	H.R.(95% CI)	*P*	H.R.(95% CI)	*P*
Age	1.00(0.99-1.01)	0.544		
Gender	1.73(0.75-3.95)	0.197		
T classification	3.53(1.77-7.01)	<0.001	2.28(1.06-4.91)	0.034
N classification	2.47(1.27-4.81)	0.008	1.95(0.95-4.04)	0.069
Distant metastasis	2.22(1.10-4.49)	0.026	1.24(0.56-2.74)	0.592
Differentiation	1.84(1.17-2.90)	0.009	1.17(0.71-1.94)	0.547
STMN1 expression	1.21(1.11-1.32)	<0.001	1.11(1.01-1.20)	0.028
Adjuvant chemotherapy	0.33(0.16-0.72)	0.005	0.43(0.19-0.94)	0.034

The STMN1 expression level and TNM stage were combined to generate a more sensitive predictive model for overall survival. ROC analysis at 36-month follow up indicated that incorporation of STMN1 expression level and TNM stage showed a better prognostic value than TNM stage alone (Figure 2E, 2F). Although the combination of TNM stage and STMN1 expression (AUC, 0.709, 95% CI 0.589-0.812) showed no significant better prognostic value than TNM stage (AUC, 0.592, 95% CI 0.468-0.708, p=0.106) alone at 12-month follow-up, while at the 36-month follow-up the integrated score (AUC, 0.755, 95% CI 0.637-0.850) revealed obviously better prognostic value compared with TNM stage (AUC, 0.637, 95% CI 0.513-0.749, p=0.0061).

### STMN1 expression and benefit from adjuvant chemotherapy (ACT)

**Figure 3:**
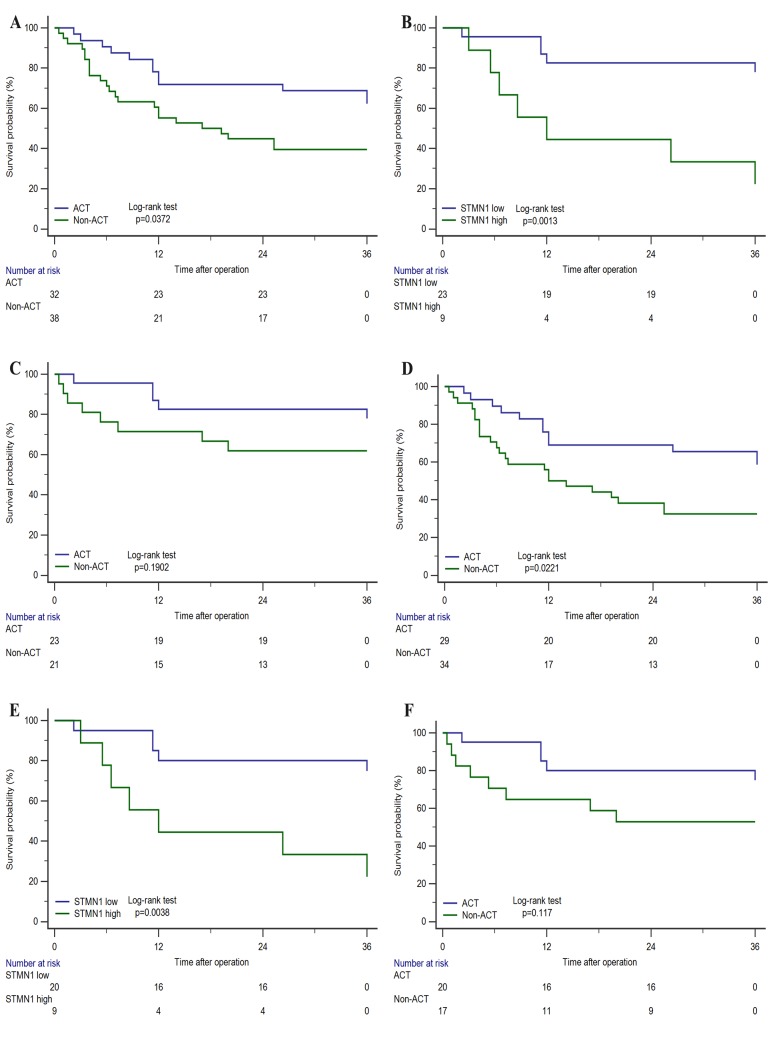
Relationship between STMN1 expression and benefit from adjuvant chemotherapy (ACT) In all patients, **(A)** ACT vs Non-ACT. **(B)** STMN1 low vs high in patients with ACT. **(C)** ACT vs Non-ACT in STMN-low patients. In TNM III- IV stage patients, **(D)** ACT vs Non-ACT. **(E)** STMN low vs high in patients with ACT. **(F)** ACT vs Non-ACT in STMN1-low patients.

To further evaluate whether patients with low STMN1 expression tumors would benefit from ACT, the relationships between STMN1 expression and overall survival of patients who received ACT or not were analyzed by Kaplan-Meier method. As shown in Figure 3, in all patients who received ACT had better overall survival compared those did not receive ACT (Figure 3A, P=0.0372). In all the patients who received ACT, those who with STMN1 low expression had longer overall survival (Figure 3B, p=0.0013). However, the use of ACT showed no significant benefits in patients with STMN1 low expression (Figure 3C, p=0.1902). Furthermore, patients with TNM III- IV stage who received ACT had better prognosis (Figure 3D, p=0.0221). In TNM III- IV stage patients, ACT could benefit STMN1 low subgroup (Figure 3E, p=0.0038). While the difference was not significant, STMN1 low patients with ACT could have better prognosis compared those without ACT (Figure 3F, p=0.117).

## DISCUSSION

STMN1, which is also known as oncoprotein 18, prosolin, can modulate microtubule dynamics through preventing polymerization of tubulin and boosting destabilization and disassembly of microtubule during the interphase and late mitosis along cell cycle progression[16–18]. Several published researches have reported that STMN1 is overexpressed in various human cancers[19, 20], including gallbladder carcinoma. Recent study demonstrated STMN1 played an important role in cell proliferation, differentiation, migration in gallbladder carcinoma, which suggested that STMN1could be a novel prognostic indicator of GBC[21–23].

In this research, we demonstrated the prognostic value of STMN1 expression in GBC and defined STMN1 expression as an independent prognostic factor for overall survival of the patients. Our finds indicated that low STMN1 expression was related with longer OS, especially in TNM III- IV stage patients with ACT. Furthermore, this predictive biomarker combined with TNM stage could stratify prognosis of GBC patients with a very high power.

Previous studies have suggested the need for additional chemotherapy following surgical resection due to the high incidence or distant recurrence of GBC[24, 25]. However, the role of adjuvant chemotherapy for GBC remains unclear and controversial[26, 27]. It is crucial to identify the patients who will get satisfactory overall outcome from ACT. Our study unraveled the relationship between STMN1 expression and prognosis in patients with ACT. We observed that no matter in all the patients or the TNM III- IV stage patients who received ACT, those who suffered from STMN1-low tumors could significantly benefit from ACT. Although no significant better outcome was found in the patients with STMN1-low who received ACT, the ACT still showed a better response in TNM III- IV stage patients with STMNI-low than in all patients with STMN1-low. This results suggested that STMN1 could be an effective predictor of adjuvant chemotherapy in TNM III- IV stage patients. And the exploration of SMTN1 in TNM III- IV stage patients may be useful for better selection and treatment of patients who should be recommended to receive ACT.

However, a few limitations should be acknowledged. First, due to this study is retrospective designed with small sample size from a single institution, it is necessary to validate these results by a large, multi-center, prospective data. Second, the exact role of STMN1 in the progression of GBC would be detected in our future work.

In conclusion, our study has identified elevated expression of STMN1 in GBC was obviously associated with unfavorable prognosis, which could be incorporated with TNM stage to generate a more precise prognostic predictive model. Moreover, the results of patients with ACT indicated that STMN1-low patients who receiving ACT tended to have improved overall survival, especially for the TNM III- IV stage patients. Therefore, assessment of STMN1 expression in GBC patients might provide direction in postoperative management for clinicians.

## MATERIALS AND METHODS

### Patients

The study enrolled 70 patients with GBC who underwent surgical resection between January 2009 and October 2013 from Zhongshan Hospital, Fudan University (shanghai, China). All specimens from patients who had been informed of the consent approved by the Clinical Research Ethics Committee of Zhongshan Hospital. All tumors were diagnosed as a primary tumor arising from the gallbladder. We retrospectively collected the clinical pathological and baseline demographic characteristics of the patients, including age, gender, tumor differentiation, tumor TNM stage. The tumor TNM stage assessment was given by two independent pathologists from Department of Pathology, Zhongshan Hospital, according to the 7th edition of UICC/AJCC cancer staging manual. Patients with adjuvant chemotherapy received at least one cycle of ACT. Follow-up data were achieved in all cases, ranging from 2 months to 72 months with a median follow-up time of 38 months. Overall survival was defined as the time from the date of surgery to the date of death or last visit.

### Tissue microarray, immunohistochemical staining

Tissue microarrays (TMA) were constructed and stained by immunohistochemical at once mentioned previously, and the tissue microarray was established with formalin-fixed paraffin-embedded specimens. Primary anti-stathmin 1 antibody (1:600; No.4191 Rabbit mAb, Cell Signaling Technology, MA, USA) was applied for immunohistochemistry staining. Membranous and/or cytoplasmic staining of the tumor cells was considered as positive. Two pathologists evaluated the staining scores using the semi-quantitative immunoreactivity scoring system, which was on a scale of 0-300, multiplying the percentage of positive tumor distribution (0-100%) by the score of staining intensity (where 3, 2, 1, and 0 indicate strong, moderate, weak, and negative staining, respectively). The cut-off point score for the definition of high/low expression subgroups were determined by X-tile software.

### Statistical analysis

SPSS 22.0 (SPSS Inc., Chicago, IL) and MedCalc Software (version 15.2.2; MedCalc, MariaKerke, Belgium) were used to perform the analysis. Chi-square test, Mann-Whitney *U* test for categorical variable. The correlation between characteristics variables and STMN expression were analyzed by student’s *t* test. Survival curves were plotted using the Kaplan-Meier method and analyzed by the log-rank test. Univariate and multivariate Cox proportional hazards regression were performed for prognostic factors. Furthermore, time dependent ROC analysis was performed by adding the weighted value of the STMN expression to the TNM stage. The data analyses were performed by MedCalc software and SPSS 22.0 with 2-tailed *P*<0.05 considered statically significant.
